# Lifetime patient outcomes and healthcare utilization for Bronchopulmonary dysplasia (BPD) and extreme preterm infants: a microsimulation study

**DOI:** 10.1186/s12887-020-02037-5

**Published:** 2020-03-25

**Authors:** Sasha van Katwyk, Sajit Augustine, Bernard Thébaud, Kednapa Thavorn

**Affiliations:** 1 Ottawa Hospital Research Institute, The Ottawa Hospital, Ottawa, ON Canada; 2 School of Epidemiology and Public Health, University of Ottawa, Ottawa, ON Canada; 3 Division of Neonatology, Windsor Regional Hospital, Windsor, ON Canada; 4 Department of Pediatrics, Schulich Medicine & Dentistry, Western University, London, ON Canada; 5 Children’s Hospital of Eastern Ontario (CHEO), Ottawa, ON Canada; 6 Institute for Clinical and Evaluative Sciences (IC/ES UOttawa), Ottawa, ON Canada; 7 The Ottawa Hospital - General Campus, 501 Smyth Road, PO Box 201B, Ottawa, ON K1H 8 L6 Canada

**Keywords:** Economic evaluation, Microsimulation, Disease burden, Preterm, Bronchopulmonary dysplasia, Pediatrics

## Abstract

**Background:**

Bronchopulmonary dysplasia (BPD) is among the most severe chronic lung diseases and predominantly affects premature infants. There is a general understanding of BPD’s significant impact on the short-term outcomes however there is little evidence on long-term outcomes. Our study estimates the lifetime clinical outcomes, quality of life, and healthcare costs of BPD and associated complications.

**Methods:**

We developed a microsimulation model to estimate lifetime clinical and economic burden of BPD among extreme preterm infants (≤28 weeks gestational age at birth) and validated it against the best available Canadian data. We further estimate the cumulative incidence of major complications associated with BPD, differentiated by BPD severity and gestational age category.

**Results:**

We find, on average, patients with BPD and resulting complications will incur over CAD$700,000 in lifetime health systems costs. We also find the average life expectancy of BPD patients to be moderately less than that of the general population and significant reductions in quality-adjusted life year due to major complications. Healthcare utilization and quality of life measures vary dramatically according to BPD severity, suggesting significant therapeutic headroom for interventions that can prevent or mitigate the effects of BPD for patients.

**Conclusions:**

Our study adds a significant expansion of existing evidence by presenting the lifetime burden of BPD based on key patient characteristics. Given the extreme cost burden at the earliest stage of life and lifetime negative impact on quality of life, there is larger headroom for investment in prevention and mitigation of severe BPD than is currently available.

## Background

Bronchopulmonary dysplasia (BPD) remains the most common complication of extreme prematurity. It is associated with severe long-term co-morbidities that reduce patient life expectancy, quality of life, and increase healthcare costs [1]. While there is some evidence on the immediate cost of BPD during and immediately following birth admission [2, 3], little is known about the long-term healthcare utilization and costs associated with BPD, or how these costs differ across a population that is known to be heterogeneous in BPD severity and subsequent complications. This is a considerable oversight as the majority of extreme preterm BPD patients do survive their first year of life, yet do so at extreme risk of developing multiple severe complications that will require lifetime treatment and care [4]. BPD is particularly common among extremely preterm infants; those under 29 weeks gestational age at birth are at high risk of developing several severe chronic morbidities that are associated with BPD. Due to the combination of risk factors facing these neonates in particular, it has been challenging to provide evidence on the likely outcomes for patients based on their age and severity of BPD. It is imperative that clinicians, parents, and the health system have a clearer picture of the risks and the long-term care and quality of life implications for an extreme preterm newborn that develops BPD. Simulation models are not replacements to long-term epidemiological studies, but they are timely tools that can synthesize data and elucidate the dynamics and patterns of disease progression that can inform decision makers quickly. A model also allows for the study and evaluation of the potential impacts of various intervention strategies for BPD, while being able to integrate new evidence as is arises.

This study used a microsimulation model to estimate a lifetime clinical and economic burden of BPD among extreme preterm infants—defined as ≤28 weeks gestational age at birth—to offer decision makers information on the expected survival, quality-adjusted life years, and health system costs associated with a preterm infant who develops BPD.

## Methods

We built a microsimulation model of extreme preterm infants (gestational age less than 29 weeks) to estimate the long-term clinical and economic outcomes of patients stratified on gestational age at birth, BPD severity at 36 weeks, and highly associated complications. Our study combined the best available evidence on the BPD risk among extreme preterm infants, BPD severity-adjusted risk of developing major complications, quality of life and health system cost for each complication in order to estimate the lifetime burden associated with BPD.

We performed a targeted literature search without any restrictions on study design or a publication date. We included studies that were based on a single centre and those were based on the large national or regional neonatal databases, including (Canadian Neonatal Network (CNN), EPICure (UK), NICHD Neonatal Research Network Generic Database (USA), Vermont Oxford Network (USA), Kaiser Permanente Medical Care program (USA), Neonatal Research Network of Japan, Epipage 1 & 2 (France), AOK National Insurance entries (Germany). We used the following free-text terms: ‘bronchopulmonary dysplasia’ OR ‘BPD’ AND ‘preterm’ OR ‘premature birth’ OR ‘very low birth weight’ OR ‘extremely low birth weight’. Free-text terms were ‘long term’ AND ‘outcomes’ OR ‘follow-up’ OR ‘complications’. The bibliographies of included studies and pertinent reviews was also be hand searched for relevant studies.

There is limited population-based data on BPD status and associated complications according to even basic cohort stratifications such as gestational age or birth weight. The CNN collects nationwide real-world data and publically reports aggregated means on discharge status, mortality rates, and some complication outcomes according to patient characteristics that would be captured in a high-level chart review of the index admission [5]. CNN data is limited in its tracking of patients post-discharge, but does provide a helpful starting point on the distribution of patients discharged from hospital. The published literature provides additional neonatal patient information on discharge rates, ventilation duration, long-term complication rate, and health care utilization [2, 6, 7]. However much of the data were reported as aggregated means from small cohorts, and only one study stratifies according to BPD severity [2]. Input parameters to the model are displayed in Table 1. The dearth of information on risk-adjusted outcomes and distributions around aggregated means necessitated a simulation approach to combine data sources and estimate patient distributions across age and risk categories (in our case, BPD status).

**Table 1:** Input Parameters for Risk of Event/Complications^a^

		BPD Severity Status					
		Mild		Intermediate		Severe	
		Mean	*SE*	Mean	*SE*	Mean	*SE*
Event	2-year Clinic Visits	3.1	*1.9*	28.5	*17.1*	28.5	*17.1*
	Hospital Admissions in Year 1	3.4	*1.7*	4.3	*2.2*	7.7	*3.9*
	Hospital Admissions in Year 2	2.3	*1.2*	2.6	*1.3*	3.6	*1.8*
	Length of Hospital Stay (Year 1 & 2), days	18.8	*2.3*	8	*2.2*	10.9	*2.0*
Risk of Complication	Neuro-impairment	34.4%	*6.9%*	58.4%	*11.7%*	78.9%	*15.8%*
	ADHD^b^	8.6%	*1.7%*	5.8%	*1.2%*	6.5%	*1.3%*
	Neurodevelopmental Delay	14.3%	*2.9%*	23.4%	*4.7%*	21.5%	*4.3%*
	Asthma	35.9%	*7.2%*	35.9%	*7.2%*	34.0%	*6.8%*
	Hearing Impairment	5.7%	*1.1%*	14.3%	*2.9%*	10.5%	*2.1%*
	Low Vision	9.1%	*1.8%*	20.8%	*4.2%*	21.1%	*4.2%*
	Pulmonary Hypertension	0.0%	*0.0%*	4.7%	*0.9%*	6.5%	*1.3%*

^a^ Sources: 2,3; ^b^*ADHD* Attention Deficit Hyperactivity Disorder

A microsimulation approach was used to facilitate the creation of sample distributions of patients at birth using the age-adjusted risk of death and the risk of developing BPD. BPD status was divided into severe, moderate, and mild. While there is debate within the field about the precise thresholds to classify severity of BPD, the relevant source data we utilized distinguished mild, moderate, and severe BPD according to a fraction of inspired oxygen (FiO_2_) of 0.21, < 0.30, and ≥ 0.30 at 36 week post-menstrual age respectively [2]. We combined no BPD status with mild since there is minimal evidence suggesting a significant difference in patient outcomes between these two groups among extreme preterm infants. Available data provides mean estimates of mortality during admission and the distribution of BPD severity at 36 weeks of age and includes confidence intervals for chronic lung disease status and increased risk of death according to specific age thresholds [5]. However, the reported evidence does not allow us to directly observe the mortality rates by both scales of BPD severity and gestational age categories necessary to distinguish outcomes at the granularity needed for decision modeling. We used the available confidence intervals to bind our analysis to avoid extreme estimates, but the model requires parameters categorized according to BPD severity and gestational age at birth the disease model to sufficiently address clinical and decision questions in the future.

We performed a first-order microsimulation and generated a sample distribution of patients with an assigned BPD severity status at 36 weeks from a ranked sample selection. We then applied a different weight to a fixed mortality rate according to severity group (severe, moderate, mild) that assumed infants with more severe BPD status are more likely to die prior to formal diagnosis at 36 weeks. The starting rates were sampled from published mortality rates [5], with the weights applied over 5000 sample iterations, and then compared to the aggregated mortality rate obtained from the published literature. Weighted mortality rates that produced aggregated death rates that fell within the published confidence range were saved by the sampling algorithm, meaning as the sampling process was repeated the potential range of potential mortality rates within each BPD severity group became increasingly clustered on values that would produce aggregated rates that matched the real-world evidence. This process generated a range of potential risk-adjusted mortality rates for each BPD severity category. Using these risk-adjusted mortality rates, the ranked sampling process was repeated to draw patients by BPD severity status adjusted for mortality during admission that would skew the distribution of patients still alive at 36 weeks [8]. The result served as a prior patient distribution of BPD at birth and at 36 weeks adjusted for differential mortality risk; with the former having a disproportionately higher number of more severe cases that were more likely to not survive to discharge. The simulated patient distributions at 36 weeks were validated against the published distributions and 94% of our sampled mortality rates fell within the reported 95% confidence range [5]. We followed the same sampling process for three different gestational age groups—less than 25 weeks at birth, 25–26 weeks, and 27–28 weeks—and combined the age groups into a final population based on the CNN reported age distributions.

Birth weight was excluded as risk factor due to minimal evidence on its impact on relative risk across both axes (mortality and BPD severity). Several sub-analyses were performed to test the impact of non-Gaussian distributions around the CNN data’s reported mean. Mortality risk by BPD status was found to significantly impact the final sample distribution. We concluded, on the basis of expert opinion and the published literature [4, 6, 7], that the mortality risk is non-normally distributed by patient characteristics and is very likely to be a competing risk of BPD status at 36 weeks. Consequentially, we applied this non-Gaussian distribution of mortality risk to the final model by applying BPD-correlated ranks to the random effects model to estimate mortality and back transformed to a uniform distribution to apply to the final joint density function [9].

Using the first-order probability density function approach described above, a final patient population of 10,000 simulants were used to represent the distribution of gestational age at birth and BPD status. The population represents infants that survived to discharge. We modelled the lifetime trajectory of patients using an annual cycle. Post discharge, patients could develop major complications highly associated to preterm infants and differentially distributed according to BPD. We included the following major complications: respiratory illnesses requiring rehospitalization with variable length of stay, clinic visits for wheezing episodes, asthma exacerbations or psychiatric illnesses such as ADHD, developmental delay which can be global or specific (global developmental delay is defined in reference to infants and preschoolers, ages 0–5 years, who present with delays of 6 months or more, in two or more of the following developmental domains: gross/fine motor, speech/language, cognition, social/personal and daily living activities while specific developmental delay (SDD) refers to age inappropriate performance in a specific area), neurological impairment (cerebral palsy), hearing impairment, vision impairment, and chronic pulmonary hypertension [10]. We performed a targeted literature search to identify the risk of developing major complications according to BPD status and average age of diagnosis in order to derive time-dependent transition probabilities for each complication.

Costs and health utilities associated with each complication were applied if the infant acquired the complication, with all costs and utilities adjusted for half-cycles in the cycle they acquired the complication and the year of death. We estimate healthcare cost from a perspective of the Canada’s publicly funded healthcare system; only costs that are borne to the health system are incorporated. Cost information was taken from published Canadian studies that specified the cost burden attributed to BPD or a specific complication (see Table 2). Costs directly pertaining to BPD included initial admission, oxygen therapy, any direct long-term costs that were identified as treatment for BPD [2], and clinic visits and hospital admissions within the first two years. Our final cost estimate for burden of BPD included cost estimates for major complications linked to BPD. When applicable, costs were time-dependent to reflect changes in healthcare utilization of a patient’s lifetime. We estimated quality-adjusted life years (QALY) by multiplying the expected life expectancy and respective health utility values. There is evidence on patient health utilities for BPD patients that differentiate by severity [11] however adjusting utilities according to an underlying BPD status differentially by combinations of complications presented significant challenges without much existing evidence to validate this approach. Consequentially, we used two different health utility measures to account for differences in expected patient utility based on the combination of complications developed over their lifetime. The base case approach assigns patients a utility score according to their BPD severity (Mild being the relative utility reference) with the complication a patient develops that has the lowest relative utility estimate being applied as additional disutility. An alternative approach treated each complication acquired as compounding a patient’s disutility and were applied multiplicatively, thereby presenting a more extreme interpretation of quality of life. Costs and utilities were discounted at 1.5% in accordance with evaluation guidelines [12]. Costs are presented in 2018 Canadian dollars.

**Table 2:** Input Parameters Used for the Model

		Proportion	Mean cost (C$, 2018)	SD	Source
***Costs***					
**Preterm Infant Index Admission**	Extreme preterm (< 28 weeks)		59,508	10,473	12
**Preterm Infant Long Term Costs**	Extreme preterm (< 28 weeks)		12,884	2150	2
**BPD (By severity) -Index Admission Cost**	All BPD		105,131	18,393	3
	Mild		75,928	15,154	
	Moderate		113,892	19,504	
	Severe		141,148	27,823	
**BPD Home Care Costs**	All BPD		475	81	
**BPD Annual Costs (Post Discharge)**	All BPD		15,628	3056	
**Annual management costs for general population (or preterm infant specific) with:**					
**ADHD**	Drug Costs	30%	827	144	13
	Psychological/Behavior therapy	15%	2880	523	
	Combination (Medication + Counseling)	32%	3707	612	
	No treatment	23%	189	34	
**Asthma**			3925	709	14
**Hearing impairment**	Cost of hearing aid device	76%	1549	295	15
	One time cost of cochlear implant	24%	46,555	8376	
	Post implant follow up year 1		4411	825	
	Post implant follow up year 2		1766	285	
	Post implant follow up year 3		1255	216	
**Retinopathy**			3914	750	16
**Pulmonary Hypertension**	Total healthcare costs		18,882	3449	17
**Neuro-impairment**	Without technical assistance	80%	11,900	1976	18
	With technical assistance	20%	46,603	8791	
**Developmental Delay**	Total healthcare cost		10,534	1861	
***Utilities***					
**Preterm BPD**	Mild BPD (reference category)		1.00		19
	Intermediate BPD		0.80	0.20	
	Severe BPD		0.50	0.10	
**ADHD**	Average		0.71	0.25	20
**Asthma**	Average		0.89	0.09	21
**Hearing impairment**	Average across the severity (mild, moderate, severe hearing loss)		0.62	0.20	22
**Retinopathy Of Prematurity**	Bilateral threshold retinopathy of prematurity		0.60	0.10	23
**Pulmonary Hypertension**	Average		0.71	0.14	24
**Neuro-impairment**	Cognitive impairment in preterm sample		0.64	0.33	25
**Developmental Delay**	Average across levels		0.42	0.41	26

Note: *BPD* Bronchopulmonary dysplasia, *ADHD* Attention Deficit Hyperactivity Disorder

Population age-dependent mortality risk was derived from Statistics Canada [13]. Adjusted relative risk of mortality following discharge for extreme preterm infants was applied to all patients based on best available evidence [14]. There is currently no long-term study that observes relative risk of mortality according to BPD status, so mortality risk is assumed to vary by gestational age at birth in this model.

All results are presented as the mean probabilistic output following a second-order Monte Carlo of 10,000 iterations wherein all parameter values were varied according to their distribution. Each second-order run utilized a starting distribution of patients distributed according to their BPD status at discharge. One thousand first-order runs of the patient distribution at discharge were run for every second-order iteration. The model was developed using R (R Foundation for Statistical Computing, Vienna, Austria).

## Results

**Fig. 1:**
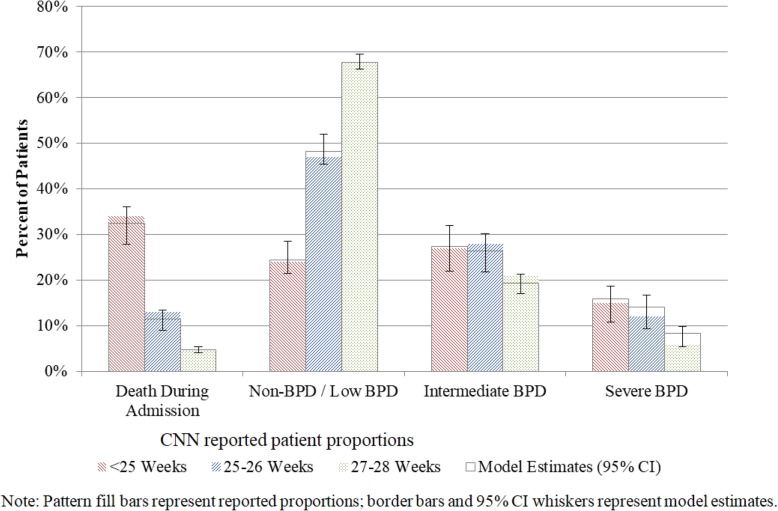
Model Comparison to CNN reported outcomes patient distribution, by gestational age

**Fig. 2:**
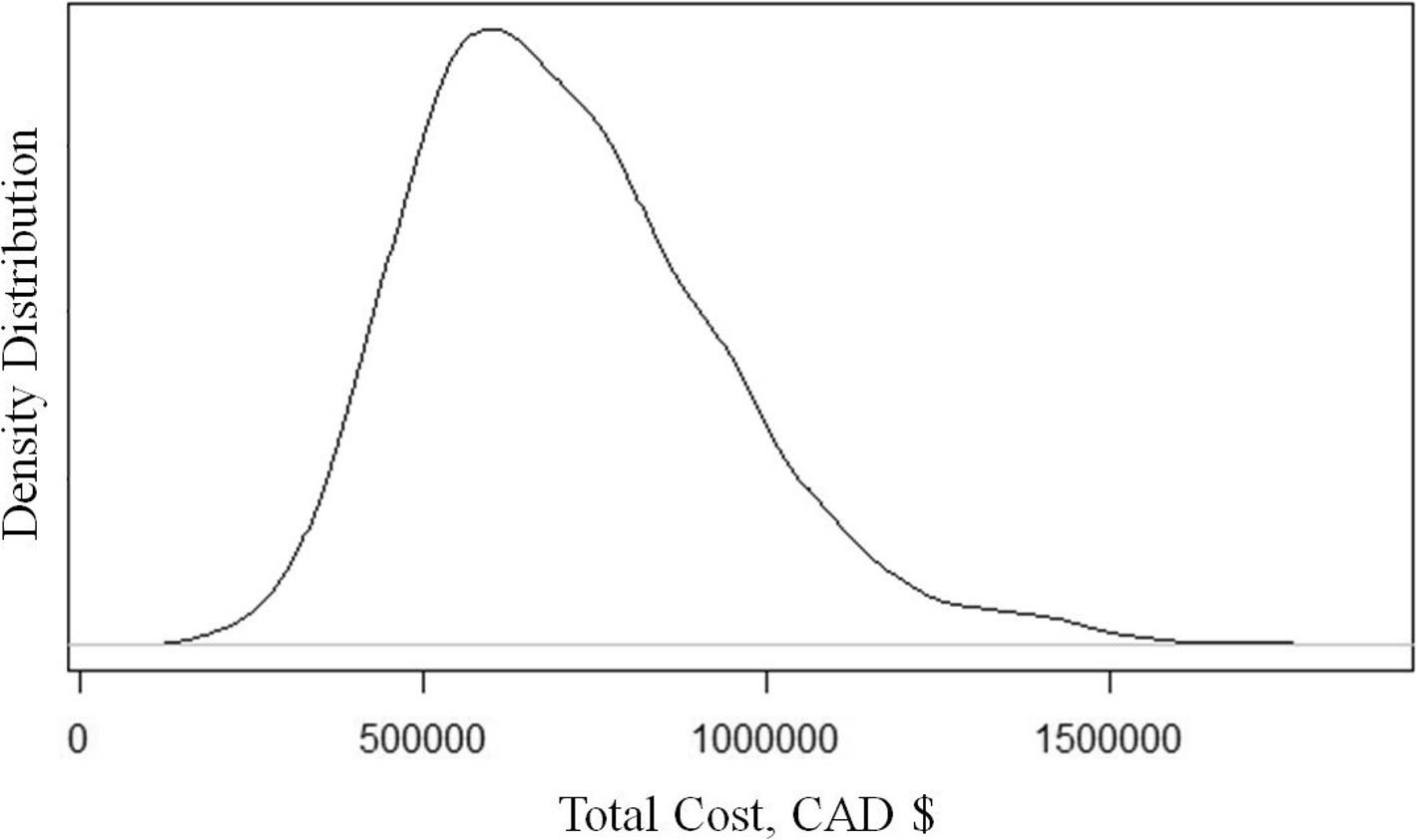
Distribution of Total Cost Estimates from all Simulations

**Fig. 3:**
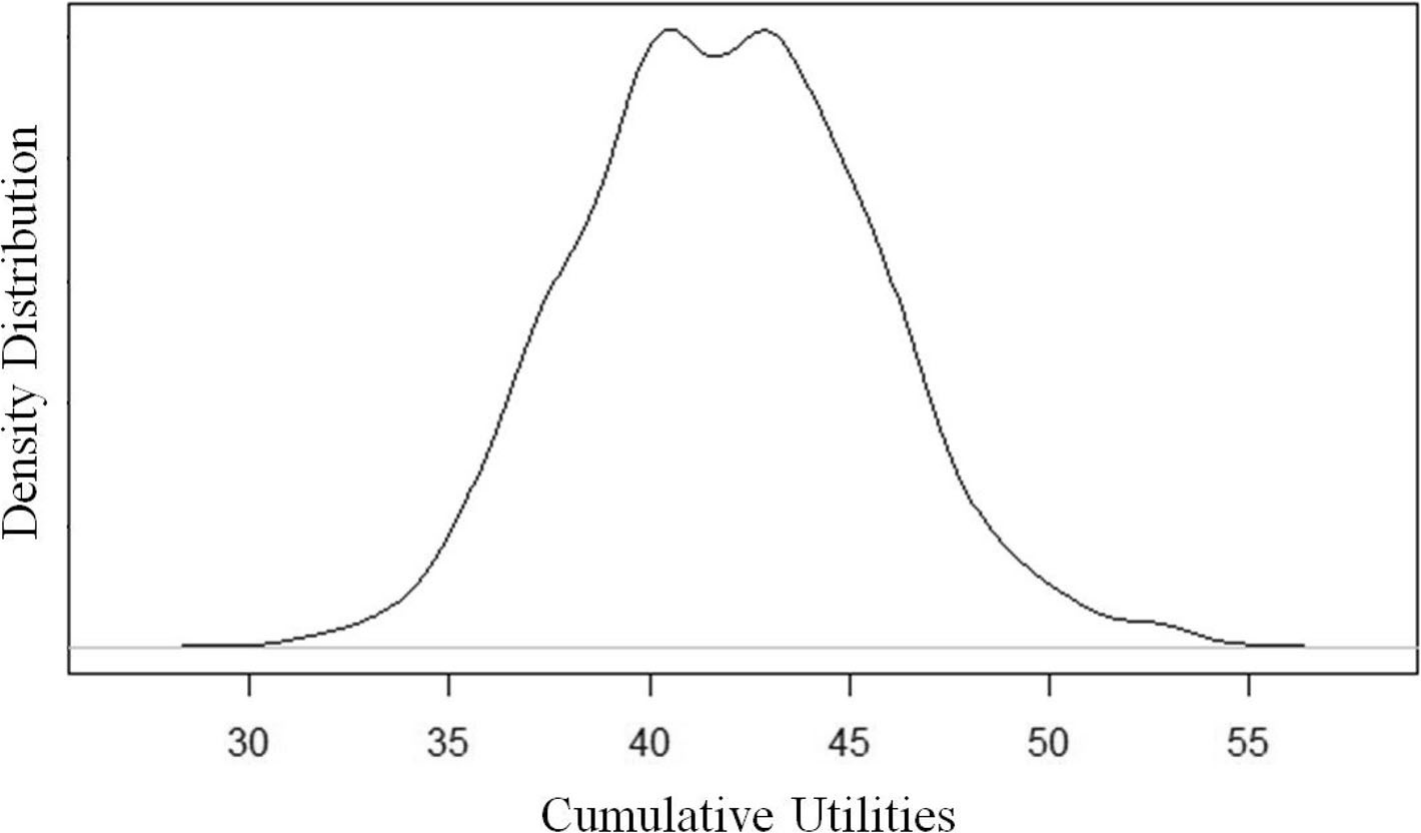
Distribution of Total Utility Estimates from all simulation runs

Our model predicts 11% of extreme preterm infants (< 28 weeks gestational age at birth) die prior to first hospital discharge, while 54, 23, and 12% of infants will be diagnosed with none/mild, intermediate, and severe BPD, respectively. Figure 1 shows the results of a validation exercise between our first-order simulation estimates and the CNN’s 2015 reported BPD status at 36 weeks of age (including mortality) [5]. The results show our estimates slightly over-estimate the proportion of patients with severe BPD, however all ‘real world’ values fall within the 95% confidence interval of our model. Figures 2 and 3 present the simulated outcome distribution of total cost and cumulative QALYs respectively.

Total lifetime expected cost among survivors to discharge is $716,912 (95% CI: $416,000 – $1,275,000), 28% ($197,100) of which is incurred in the first year. We estimate an additional 13% (90,600) of lifetime costs are incurred in the second year due to clinic visits and hospital admissions. The third year is estimated to account for 1.3% ($9560) of total costs, with a very gradual annual decline in expected costs over the patient lifetime, representing costs incurred from major complications developed over time. For patients that survive to discharge, their life expectancy is 73 years and their expected lifetime QALYs is 42.1. An alternative multiplicative method to account for compounding disutility from acquiring several complications estimates the expected lifetime QALYs to fall to 22.7.

Table 3 presents stratified outcomes according to BPD severity and gestational age at birth. The average infant born < 25 weeks of age that survives to discharge is expected to incur lifetime health system costs roughly 14% higher than that of an infant born at 27–28 weeks, and 13% lower health utilities. Comparatively, a severe BPD patient is expected to incur lifetime health system costs 69% higher than that of a non-BPD or mild BPD patient while accruing 59% lower health utility. Table 4 presents combined group outcomes by BPD severity and gestational age.

**Table 3:** Lifetime Expected Outcomes among Survivors to Discharge

		*By BPD Severity*			*By Gestational Age at Birth*		
	Total	*No/Low BPD*	*Intermediate*	*Severe*	*27–28*	*25–26*	*< 25*
**Expected Lifetime Outcomes (95% CI rounded to nearest 1000)**							
Costs	$ 716,912 (416,000 – 1,275,000)	$ 554,650 (298,000 – 960,000)	$ 865,550 (285,000 – 1,471,000)	$ 938,900 (550,000 – 1,748,000)	$ 657,994 (376,000 – 1000,000)	$ 708,416 (419,000 – 1,117,000)	$ 749,110 (531,000 – 1,379,000)
QALYs	42.1 (37.2–48.1)	50.4 (45.1–55.8)	33.2 (29.3–39.1)	20.1 (16.0–22.5)	43.8 (34.9–48.1)	40.6 (34.7–46.4)	37.4 (31.1–41.1)
**Expected Complication Prevalence**							
Neuro-Impairment	47%	34%	59%	79%	45%	49%	53%
ADHD	8%	9%	6%	6%	8%	8%	7%
Neuro-Development Delay	18%	14%	24%	22%	17%	18%	19%
Asthma	36%	36%	36%	34%	35%	36%	36%
Hearing Impairment	9%	6%	15%	10%	8%	9%	10%
Retinopathy	14%	9%	21%	21%	13%	15%	16%
Pulmonary Hypertension	2%	0%	4%	7%	2%	2%	3%

Note: *BPD* Bronchopulmonary dysplasia; *ADHD* Attention Deficit Hyperactivity Disorder

**Table 4:** Lifetime Expected Costs and QALYs, by BPD Severity and Gestational Age

			*By BPD Severity*		
			*No/Low BPD*	*Intermediate*	*Severe*
*By Gestational Age at Birth*	*27–28*	Costs (95% CI)	$ 510,440 (288,000 - 779,000)	$557,210 (344,600 - 821,000)	$589,993 (411,300 - 1000,000)
		QALYs (95% CI)	47.0 (37.1–51.9)	38.1 (30.3–47.4)	29.7 (15.2–46.6)
	*25–26*	Costs (95% CI)	$ 806,641 (311,000 - 835,000)	$ 869,530 (451,000 - 1,090,000)	$ 919,479 (613,000 - 1,500,000)
		QALYs (95% CI)	44.4 (33.0–49.7)	36.7 (29.9–43.0)	28.1 (14.8–44.4)
	*< 25*	Costs (95% CI)	$ 876,086 (392,000 - 960,000)	$ 943,217 (562,000 - 1,114,000)	$ 997,399 (733,000 - 1,809,000)
		QALYs (95% CI)	42.7 (28.5–47.3)	34.3 (21.3–39.9)	24.8 (13.9–38.1)

## Discussion

There are a small number published trials and observational studies that partially describe the BPD distribution of extreme preterm infants and the relative burden of BPD [1–3, 11, 15–17]. Our study adds a significant expansion of existing evidence by presenting the lifetime burden of BPD based on key patient characteristics. We have shown that the present value of the lifetime expected burden of a BPD patient discharged in 2018 is over $700,000 to the healthcare system. Excluding the high costs incurred in the first year, BPD costs the health system $4600 per patient per year, making it roughly twice as expensive as type II diabetes annually [18, 19] while the BPD cost is incurred at the very start of a patient’s life. Moreover, the expected QALYs of the average BPD patient is only 42, amounting to an annual expected quality of life score of less than 0.58 (1 indicating perfect health and 0 indicating death). This utility measure may be nearly halved depending on how one accounts for compounding disutility from multiple complications.

This study attributes major preterm complications as part of the burden pathway of BPD, largely to illustrate the association between BPD as an early clinical marker and risk factor for many clinical outcomes resulting from extreme preterm birth. There are several major complications that were not included in our model primarily because we only included complications for which previous literature outlined a differential incidence of complications associated with BPD severity. This model is not meant to reflect the total burden for the average preterm infant, as they may develop complications that are not associated with BPD severity and/or were not captured in the existing BPD literature.

An additional benefit from our simulation model is we now have valuable insight into the scale and shape of the distributions around our prevalence estimates. This is often missing in other model outputs and the vast majority of evidence available to date is based on single sources or time periods, lending no opportunity to apply a range or shape and to characterize the uncertainty around the mean. Figures 2 and 3 present the distributions for the whole population for cost and health utilities obtained from our study, respectively. The cost distribution has an unsurprising right-tail skew indicating a high potential of extreme costs incurred by a portion of patients that pulls the average cost per patient higher. The Additional file 1 presents several smoothed histograms to show the density of values across all simulants averaged across all runs, as well as the survival curve for all patients that survived to discharge.

The sampling approach used here intentionally applied very little constraint on the sample distribution to allow for our results to be as conservative as possible in attributing high uncertainty to our estimates. Our findings ended up being well aligned to the CNN reported data despite the only use of the CNN data was to assign the rank order of discharge outcomes and to test the bootstrapped samples relative to the CNN reported aggregate mortality confidence range.

There have been well documented attempts at predicting BPD outcome based on gestational age and weight at birth as well as functional outcomes in the first days of life, however these models are limited to predicting BPD severity in the short term and do not focus on long-term outcomes or risk of developing complications as a result of BPD status [20]. Large scale longitudinal studies that would elucidate the long-term impacts of BPD are strongly encouraged in order to validate our findings. We recognize such a study would be a more time and resource intensive than our simulation approach, however based on the magnitude of health and cost burden BPD appears to present, we do believe there is value in a more intensive observational study.

Not surprisingly, our study shows that expected costs, QALYs, and complication rate vary significantly by gestational age at birth or BPD severity. The published literature presented complication prevalence by BPD severity. Our study adds a new contribution to the existing literature by revealing the long-term clinical and economic outcomes by gestational age and BPD severity. A crucial question that remains is whether expected costs and QALYs in fact present as forecasted or if there are latent costs and effects on life expectancy that are expressed later in life, beyond what observational data currently captures. Long-term costs and health utilities for particularly severe complications are likely to be associated with higher risk of death, suggesting some simulants of this model that have severe complications and/or multiple complications have over-estimated costs (due to near end of life care currently unaccounted for) and quality of life measures. It is our hope that further longitudinal research can elucidate the risk-adjusted mortality rate of patients in order to provide better information on disease and care trajectories of BPD. Our simulation model can be extended to test what relative risk increase in mortality would be necessary to see significant changes in our outcome estimates, however this is better explored in the context of an economic evaluation of treatments for BPD.

The best available literature using the same Canadian health system perspective on the long-term healthcare utilization for BPD patients is a 2008 paper by Landry et al. where they estimate the cost of BPD per patient to be $15,700 (adjusted to 2018 dollars) for the first 20 years, excluding index admission. In comparison, our model estimates the annual cost per patient is $16,500 over the same time period, excluding index admission. While closely matched, the two estimates derived costs differently. Landry et al. attributed all hospitalizations and pharmaceutical fees by BPD patients while not explicitly incorporating costs of major complications. In comparison, our study attributed the first 2 years of hospitalizations to BPD and then based costs on annual costs of major complications, which would include hospitalizations, pharmaceuticals, and reimbursable material costs [18, 21–33].

Evidence on short- to medium-term burden of BPD from non-Canadian sources reinforces our study’s findings about the extreme first 2 years’ cost of BPD followed by consistent costs incurred as a consequence of major complications [1] and scale of cost difference according to BPD severity. For instance, Álvarez-Fuente et al. found the cost for the most severe BPD cases was roughly 50% higher in the first 2 years than other BPD patients [1]; in comparison our model estimated 44% higher cost of severe BPD cases compared to other patients. While it is difficult to directly compare costs across different health systems and patient groups (Álvarez-Fuente et al. observed all preterm infants rather than extreme preterm), it is encouraging to find differential scales of burden according to severity of BPD. Our study advances the evidence based in projecting long-term health outcomes and cost implications, as well as explicitly incorporating major complications to provide clinical detail on the patient distribution and differential effects on lifetime burden of BPD.

This study focuses on lifetime burden of patients within a Canadian health system perspective, presenting some limitations to generalizability under different health system regimes. The precise costs and outcomes for patients will obviously change under different systems of care, however we believe that the broader implications of health outcomes and the incidence of adverse events will not significantly be different in non-Canadian settings. Our study can therefore provide a starting point for a more general estimation of expected burden of care in other settings.

Due to the limitations regarding data on long-term mortality risk among BPD patients, life expectancy and survival curves were not included as primary outcomes of the model at this stage. While we did incorporate a relative risk to the general population mortality rate based on the best evidence for extreme preterm infants [14], this is non-differential across gestational age at birth or BPD status. Additionally, our model does not yet include risk of mortality associated with major complications, which we would expect to impact survival. Consequentially, our life expectancy estimates are likely to be over-estimated. While this has minimal effect on the total cost estimate since the majority of costs are incurred earlier in life, our health utility estimates are correlated with life expectancy and will be over-estimated concurrent to life expectancy after adjusting for utility discounting.

A limitation of our simulation approach is that the initial population of patients is based on a first-order probability density function approach. While the sampling method provided BPD severity distributions that closely resembled real-world evidence, it did not incorporate other patient characteristics such as birth weight and other perinatal conditions that may be important to accurately predicting adjusted mortality and complication risks. While it is important for these factors to be accounted for in future models, we believed it was important to have an initial model that was based on a smaller number of risk factors—in our case, gestational age at birth and BPD severity—to minimize the number of sources of structural uncertainty in our model. In the absence of clear etiological relationships between correlated risk factors, it is difficult to validate whether a simulated biological pathway is genuine—a risk that increases as more complex interactions across several risk factors are introduced to the model [17]. For the purposes of describing the burden of BPD, we believe that gestational age is the primary contributor to differential BPD severity distributions within the extreme preterm population as it is highly correlated to birth weight and other functional outcomes.

Another limitation of this study is that the long-term mortality risk for patients is only based on a long-term longitudinal study of preterm infants, which reports adjusted mortality risk according to extreme preterm birth status (< 28 weeks gestational age at birth) but no other risk factors. This is a limitation due to this model being specifically designed to describe differential outcomes among BPD patients, yet mortality outcomes are assumed to be constant across severity strata. We would expect that mortality risk would differ according to BPD severity however there is currently no evidence to establish this. Additionally, better evidence may find that BPD severity is not the predominant factor and that instead other differential risk factors such as early lung function and major complications are better predictors of mortality risk. Our model is capable of incorporating such evidence, however given the limited evidence currently available this remains an under-developed area of the model.

Finally, our model assumes that the risk of complication is independent of other complication status except for BPD severity. The same joint distribution of random effects model from the first stage of our model was applied to estimate the risk of complications after controlling for the risk of mortality. A variance-covariance matrix on the relative risk of complication dependent on other complication status was derived to adjust for compounding risk factors however without sufficient cross-correlation data from the published evidence imputation attempts introduced too much variability into the model to be useful.

Our findings highlight the predicted risks and the long-term health care needs for extreme preterm infants (< 28 weeks gestational age at birth) given the current standard of care in Canada. Infants who are discharged are expected to have a reasonably high life expectancy, however the high risk of major complications positively correlated with BPD severity results in severe reductions in expected quality of life. Given the extreme cost burden at the earliest stage of life and lifetime negative impact on quality of life, the most promising interventions would be prevention or mitigation of BPD’s effects that result in the most severe forms of chronic lung disease in extreme preterm infants. Our model and study findings can be used to estimate the maximum scope for therapeutic or health system benefits of a new BPD treatment relative to other existing treatments. The model could also inform research and development decisions and help identify patient and intervention characteristics that will make new treatments for BPD reimbursable.

## Supplementary information

**Additional file 1: Appendix Figure 1**: Distribution of Total QALY Estimates from all simulation runs using multiplicative disutility count.

**Additional file 2: Appendix Figure 2**: Survival Curve of General Canadian Population vs. Extreme Preterm Survivors to Discharge.

**Additional file 3: Appendix Figure 3**: Density of Individual Simulant Lifetime Costs.

**Additional file 4: Appendix Figure 4**: Density of Individual Simulant Lifetime QALYs.

## Data Availability

The datasets during and/or analysed during the current study available from the corresponding author on reasonable request.

## Abbreviations

ADHD: Attention Deficit Hyperactivity Disorder
BPD: Bronchopulmonary dysplasia
CNN: Canadian Neonatal Network

## References

[1] Álvarez-Fuente M, Arruza L, Muro M (2017). The economic impact of prematurity and bronchopulmonary dysplasia. Eur J Pediatr.

[2] Landry JS, Chan T, Lands L, Menzies D (2011). Long-term impact of Bronchopulmonary dysplasia on pulmonary function. Can Respir J.

[3] Landry JS, Croitoru D, Jin Y, Schwartzman K, Benedetti A, Menzies D (2012). Health care utilization by preterm infants with respiratory complications in Quebec. Can Respir J.

[4] Deakins KM (2009). Bronchopulmonary dysplasia. Respir Care.

[5] The Canadian Neonatal Network Annual Report 2017 <http://www.canadianneonatalnetwork.org/Portal/LinkClick.aspx?fileticket=XhPMIxFgc2M%3d&tabid=39>. Accessed 9 July 2019.

[6] McEvoy CT, Aschner JL (2015). The natural history of Bronchopulmonary dysplasia: the case for primary prevention. Clin Perinatol.

[7] Stoll BJ, Hansen NI, Bell EF (2015). Trends in care practices, morbidity, and mortality of extremely preterm neonates, 1993-2012. JAMA..

[8] Zucchelli E, Jones A, Rice N (2012). The evaluation of health policies through dynamic microsimulation methods. Int J Microsimulation.

[9] Elston RC, Olsen JM, Palmer L (2002). Biostatistical genetics and genetic epidemiology.

[10] American Psychiatric Association (2013). The diagnostic and statistical manual of mental disorders.

[11] Petrou S, Abangma G, Johnson S, Wolke D, Marlow N (2009). Costs and health utilities associated with extremely preterm birth: evidence from the EPICure study. Value Health.

[12] Guidelines for the economic evaluation of health technologies: Canada. 4th ed. Ottawa: CADTH; 2017. https://www.cadth.ca/dv/guidelines-economic-evaluation-health-technologies-canada-4th-edition.

[13] Shumanty, R Report on the Demographic Situation in Canada: Mortality 2014-2016. Statistics Canada 2015; Accessed: 1 Sept 2018 <https://www150.statcan.gc.ca/n1/pub/91-209-x/2018001/article/54957-eng.htm>.

[14] Swamy GK, Ostbye T, Skjaerven R (2008). Association of preterm birth with long-term survival, reproduction, and next-generation preterm birth. JAMA..

[15] Johnston KM, Gooch K, Korol E (2014). The economic burden of prematurity in Canada. BMC Pediatr.

[16] Lapcharoensap W, Lee HC, Nyberg A, Dukhovny D (2018). Health care and societal costs of Bronchopulmonary dysplasia. NewReviews..

[17] Pitman R, Fisman D, Zaric GS (2012). With ISPOR-SMDM modeling good research practices task force. Dynamic transmission modeling: a report of the ISPOR-SMDM modeling good research practices task force working Group-5. Med Decis Mak.

[18] Goeree R, Lim ME, Hopkins R (2009). Prevalence, total and excess costs of diabetes and related complications in Ontario, Canada. Can J Diabetes.

[19] Rosella LC, Lebenbaum M, Fitzpatrick T (2015). Impact of diabetes on healthcare costs in a population-based cohort: a cost analysis. Diabet Med.

[20] Trembath A, Laughon MM (2012). Predictors of bronchopulmonary dysplasia. Clin Perinatol.

[21] Miller A, Lee S, Raina P, Klassen A, Zupancic J, Olsen L (1999). A review of therapies for attention-deficit/hyperactivity disorder.

[22] Polisena J, Tam S, Lodha A, Laporte A, Coyte PC, Ungar WJ (2007). An economic evaluation of asthma action plans for children with asthma. J Asthma.

[23] Fitzpatrick E (2006). Economic evaluation of Cochlear implants in children. J Speech-Language Pathol Audiol.

[24] Lee SK (2001). Evidence for changing guidelines for routine screening for retinopathy of prematurity. Arch Pediatr Adolesc Med.

[25] Coyle K, Coyle D, Blouin J (2016). Cost effectiveness of first-line Oral therapies for pulmonary arterial hypertension: a Modelling study. PharmacoEconomics.

[26] Cohen E, Berry JG, Camacho X, Anderson G, Wodchis W, Guttmann A (2012). Patterns and costs of health care use of children with medical complexity. Pediatrics..

[27] Matza LS, Secnik K, Rentz AM (2003). PMH23 Development and Assessment Of Health State Utilities For Attention Deficit/Hyperactivity Disorder In Children Using Parent Proxy Report. Value Health.

[28] Juniper EF, Guyatt GH, Feeny DH, Griffith LE, Ferrie PJ (1997). Minimum skills required by children to complete health-related quality of life instruments for asthma: comparison of measurement properties. Eur Respir J.

[29] Smith-Olinde L, Grosse SD, Olinde F, Martin PF, Tilford JM (2008). Health state preference scores for children with permanent childhood hearing loss: a comparative analysis of the QWB and HUI3. Qual Life Res.

[30] Palmer EA, Flynn JT (1992). Cryotherapy for treatment of threshold retinopathy of prematurity. Retinopathy of prematurity.

[31] Shafazand S, Goldstein MK (2004). Health-related quality of life in patients with pulmonary arterial hypertension. Chest..

[32] Petrou S, Johnson S, Wolke D, Hollis C, Kochhar P, Marlow N (2010). Economic costs and preference-based health-related quality of life outcomes associated with childhood psychiatric disorders. Br J Psychiatry.

[33] Rosenbaum P (2007). Quality of life and health-related quality of life of adolescents with cerebral palsy. Dev Med Child Neurol.

