# Supplementary figures and images for: Lifetime patient outcomes and healthcare utilization for Bronchopulmonary dysplasia (BPD) and extreme preterm infants: a microsimulation study
Source: BMC Pediatr. 2020 Mar 25;20:136. doi: 10.1186/s12887-020-02037-5 (PMC7093972; doi:10.1186/s12887-020-02037-5)

Density Distribution

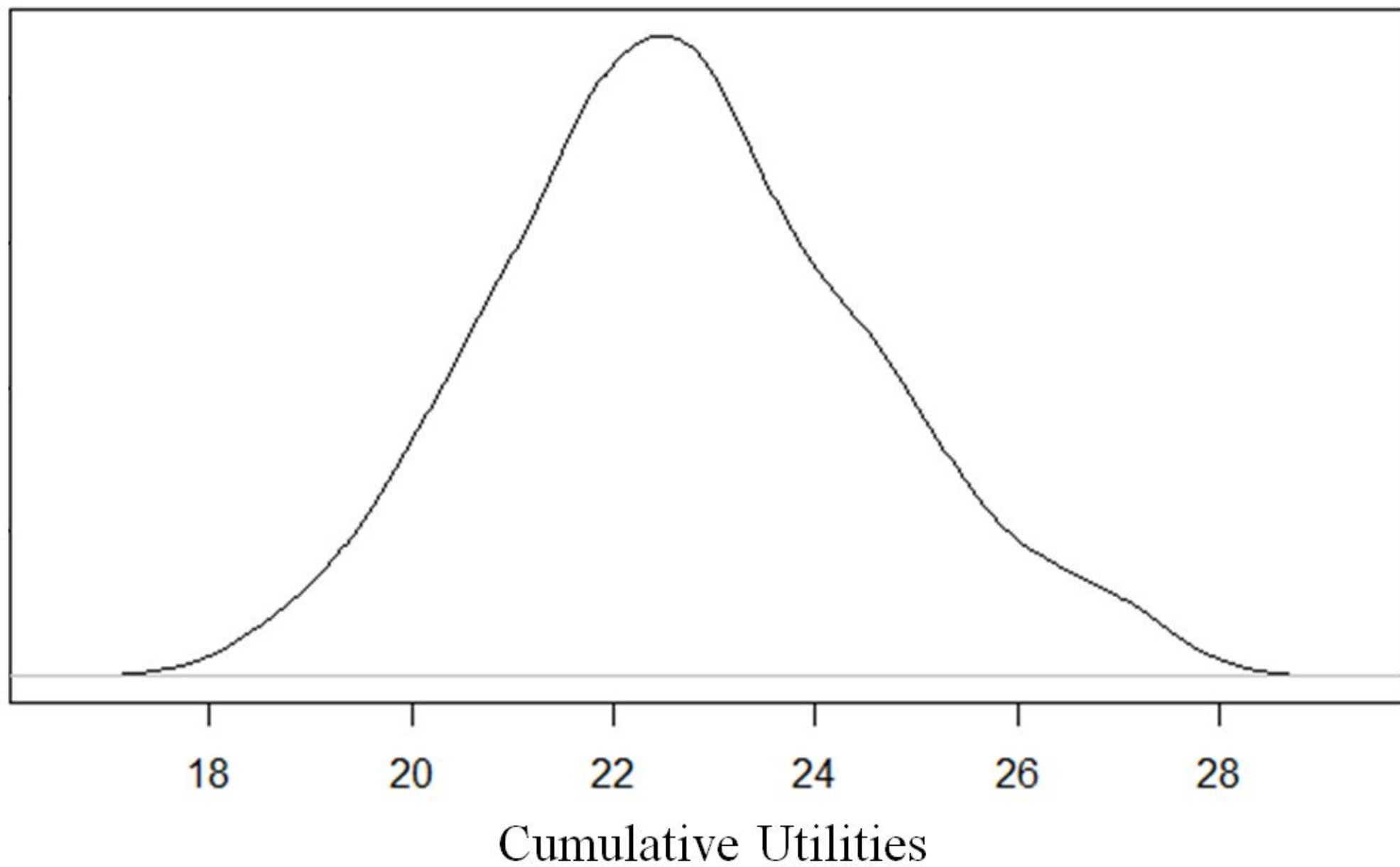

Supplement: Supplementary file 1 — Additional file 1: Appendix Figure 1: Distribution of Total QALY Estimates from all simulation runs using multiplicative disutility count. [file 12887_2020_2037_MOESM1_ESM.pdf]

## Survival Comparison, by Cohort vs General Pop

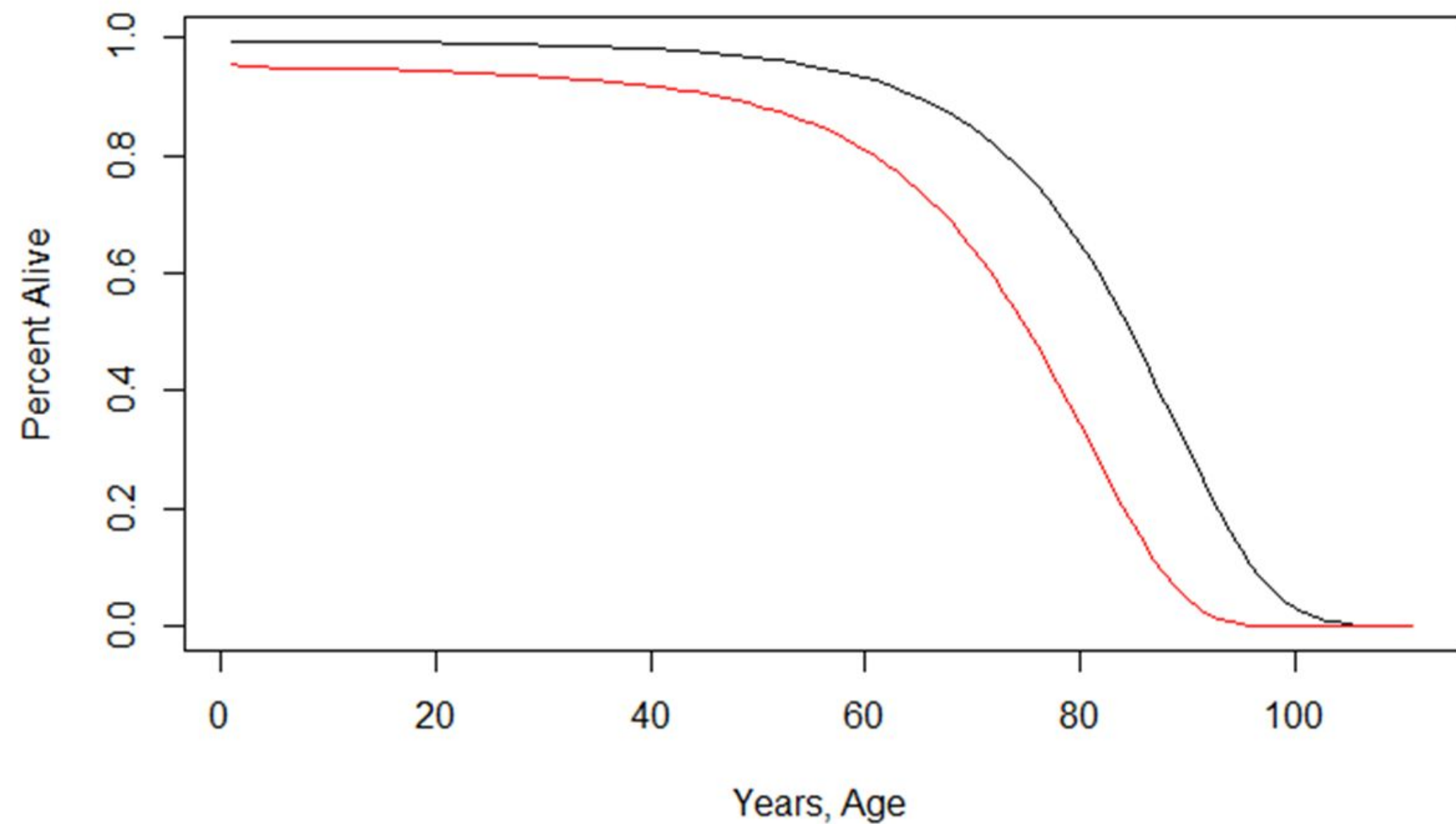

Supplement: Supplementary file 2 — Additional file 2: Appendix Figure 2: Survival Curve of General Canadian Population vs. Extreme Preterm Survivors to Discharge. [file 12887_2020_2037_MOESM2_ESM.pdf]

Frequency Distribution

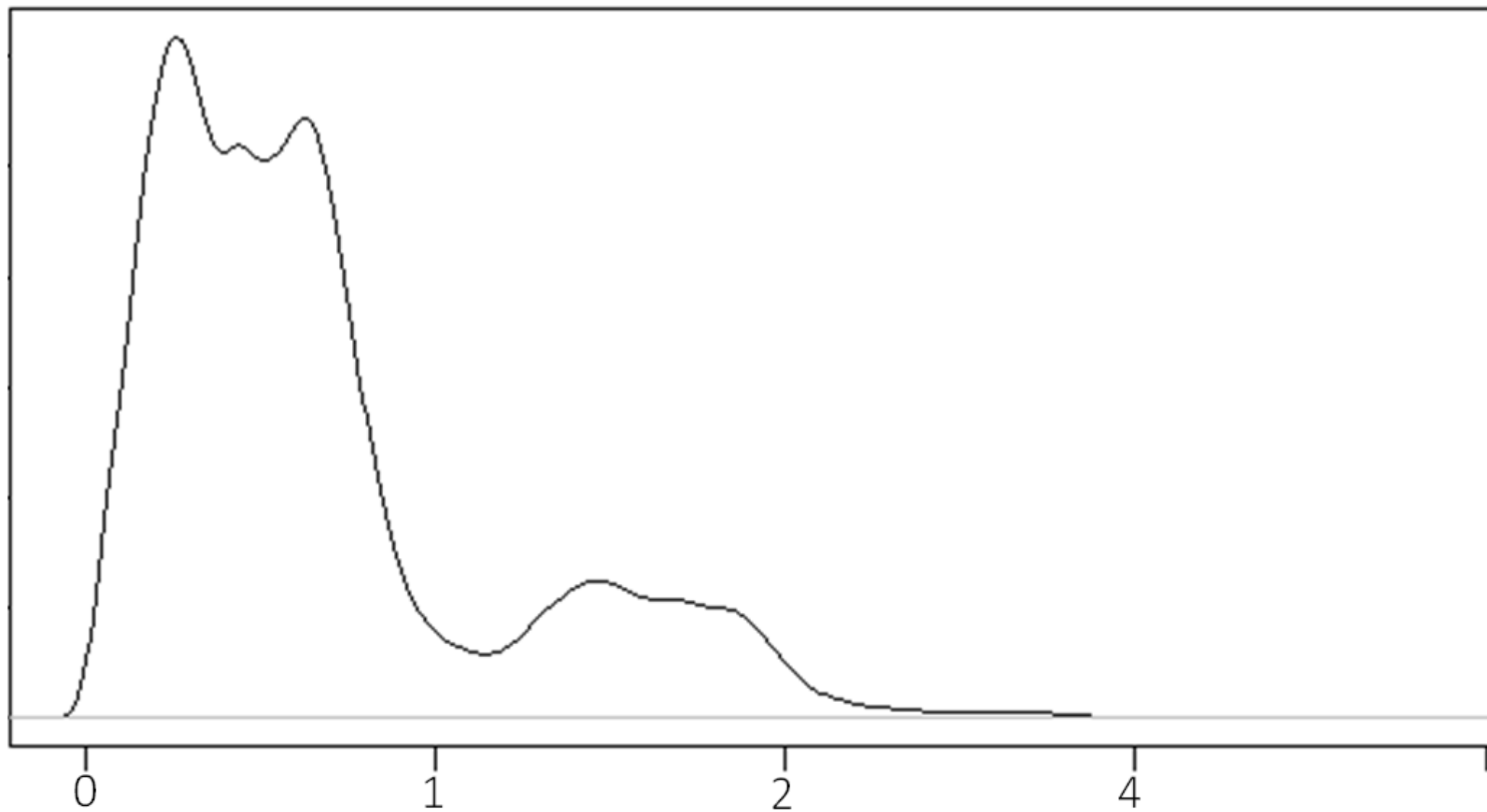

Per Person Cost, CAD \$ millions

Supplement: Supplementary file 3 — Additional file 3: Appendix Figure 3: Density of Individual Simulant Lifetime Costs. [file 12887_2020_2037_MOESM3_ESM.pdf]

Frequency Distribution

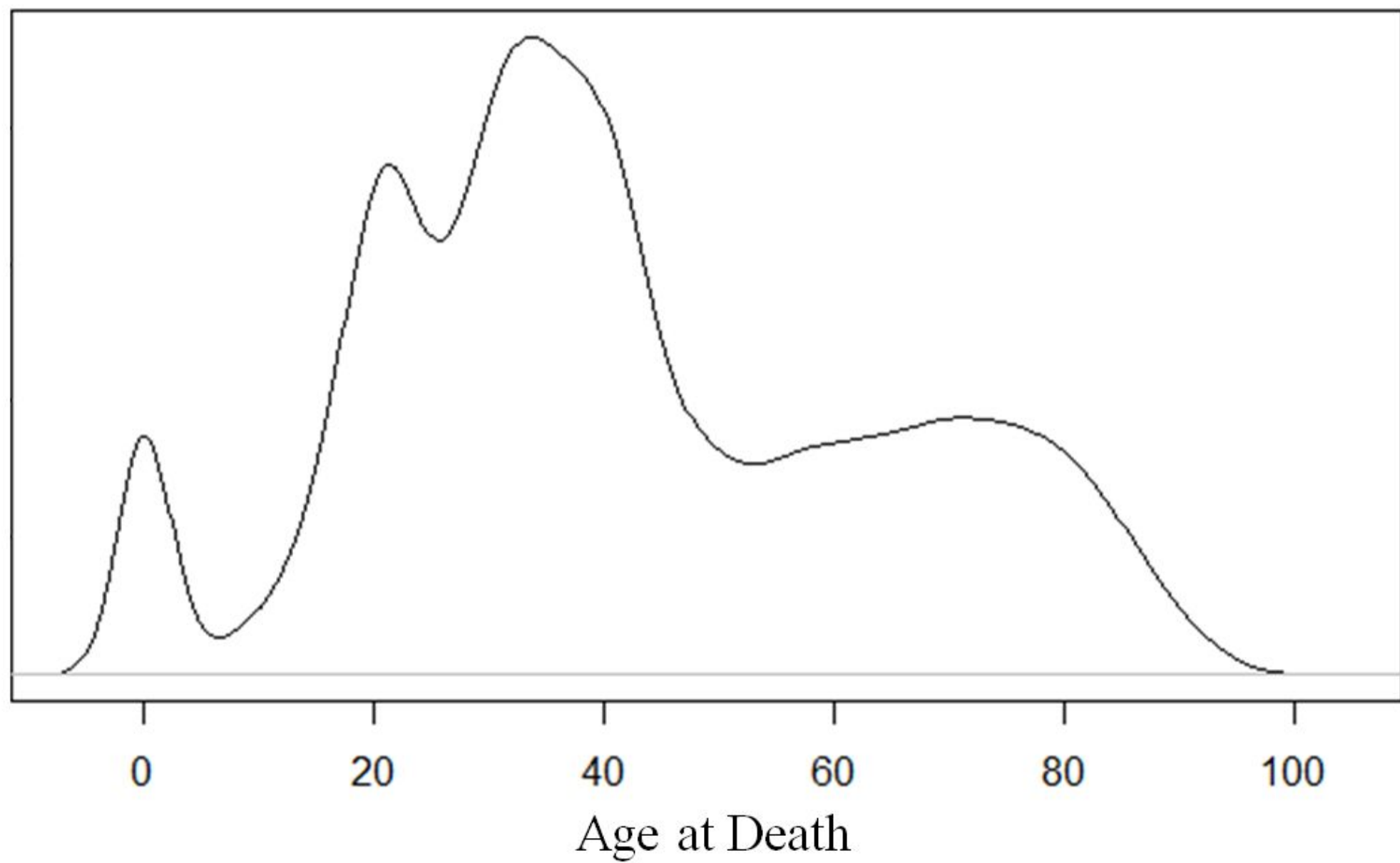

Supplement: Supplementary file 4 — Additional file 4: Appendix Figure 4: Density of Individual Simulant Lifetime QALYs. [file 12887_2020_2037_MOESM4_ESM.pdf]
